# Efficacy of chloroquine for the treatment of *Plasmodium vivax* in the Saharan zone in Mauritania

**DOI:** 10.1186/s12936-015-0563-0

**Published:** 2015-01-28

**Authors:** Mohamed Salem Ould Ahmedou Salem, Yeslim Ould Mohamed Lemine, Jemila Mint Deida, Mohamed Aly Ould Lemrabott, Mohamed Ouldabdallahi, Mamadou dit Dialaw Ba, Ali Ould Mohamed Salem Boukhary, Mohamed Lemine Ould Khairy, Mohamed Boubacar Abdel Aziz, Pascal Ringwald, Leonardo K Basco, Saidou Doro Niang, Sidi Mohamed Lebatt

**Affiliations:** Unité de Recherche « Génome et Milieux », Faculté des Sciences et Techniques, Université des Sciences, de Technologie et de Médecine, Nouveau Campus Universitaire, BP 5026 Nouakchott, Mauritania; Ministère de la Santé, Nouakchott, Mauritanie; Initiative mauritanienne pour la lutte contre les maladies endémiques « MEDCINGO », ilôt 358, Riyad Pk8, Nouakchott, Mauritania; World Health Organization Country Office, Ilot K140-141, Route de la corniche ouest, Tevragh Zeina, B.P. 320 Nouakchott, Mauritania; Drug Resistance and Containment, Global Malaria Programme, World Health Organization, 20 Avenue Appia, 1211, Geneva, 27 Switzerland; Unité Mixte de Recherche 198, Unité de Recherche des Maladies Infectieuses et Tropicales Emergentes, Institut de Recherche pour le Développement, Faculté de Médecine La Timone, Aix-Marseille Université, Marseille, France

**Keywords:** Drug resistance, *Plasmodium vivax*, Artemisinin, 4-aminoquinolines, Clinical trial

## Abstract

**Background:**

In 2006, the Mauritanian Ministry of Health adopted a new therapeutic strategy based on the systematic use of artemisinin-based combination therapy (ACT), artesunate-amodiaquine and artemether-lumefantrine, for the first- and second-line treatment of uncomplicated malaria, respectively, regardless of *Plasmodium* spp. In the Saharan zone of the country, recent studies have shown that *Plasmodium vivax* largely predominates over *Plasmodium falciparum*. Anti-malarial drug response of *P. vivax* has not been evaluated in Mauritania. The aim of the present study was to evaluate the clinical efficacy and tolerance of chloroquine to treat *P. vivax* malaria in Mauritanian patients.

**Methods:**

*Plasmodium vivax*-infected patients aged > 6 months old were enrolled in Nouakchott and Atar in September–October 2013. Chloroquine was administered at the standard dose of 25 mg base/kg body weight over three days. Patients were followed until day 28, according to the standard 2009 World Health Organization protocol.

**Results:**

A total of 128 patients (67 in Nouakchott and 61 in Atar) were enrolled in the study. Seven patients (5.5%) were either excluded or lost to follow-up. Based on the per protocol analysis, chloroquine efficacy (adequate clinical and parasitological response) was 100%. Treatment was well-tolerated. One patient was excluded on day 1 due to urticaria and treated with artesunate-amodiaquine.

**Conclusions:**

Although the current national treatment guideline recommends artesunate-amodiaquine for the first-line treatment of uncomplicated malaria, including *P. vivax* malaria, chloroquine may still have an important role to play in anti-malarial chemotherapy in Mauritania. Further epidemiological studies are required to map the distribution of *P. vivax* and *P. falciparum* in the country.

## Background

Malaria is one of the major public health problems in Mauritania. Every year, between 200,000 and 300,000 malaria cases are officially notified, mostly without laboratory confirmation of clinical diagnosis [1]. Most *Plasmodium falciparum* infections occur in the sahelian south of Mauritania. In contrast, recent epidemiological data indicate that *Plasmodium vivax* infections predominate in the Saharan zone of the country [2,3].

The epidemiology of *P. vivax* malaria is not well known in the neighbouring countries, such as Mali and Senegal. Malaria risk due to *P. vivax* is limited in Algeria [4]. *Plasmodium vivax* was eliminated from Morocco in 2010 [5]. Although *P. vivax* had been registered in the past in Cape Verde islands, only *P. falciparum* has been diagnosed during the last 20 years [6]. Elsewhere in Africa, *P. vivax* occurs in São Tomé and Príncipe, in some East African countries, and in Madagascar [7-9].

Chloroquine-resistant *P. falciparum* is widespread in West Africa [10]. As a consequence, most West African countries have adopted artemisinin-based combination therapy (ACT) for the treatment of acute uncomplicated falciparum malaria. Since 2006, in line with the regional anti-malarial drug policies, the Mauritanian Ministry of Health recommends the use of artesunate-amodiaquine and artemether-lumefantrine for the first- and second-line treatment of acute uncomplicated malaria, respectively, without making a distinction between different malaria species. Although ACT has been used to treat mixed *P. falciparum* and *P. vivax* infections in endemic areas, where mixed infections with these two *Plasmodium* species occur frequently, the efficacy of different forms of ACT to specifically treat *P. vivax* infections is not well established.

In other parts of the world, where *P. vivax* is endemic, chloroquine is still the drug of choice to treat this infection [11]. Chloroquine is generally effective against *P. vivax*, well-tolerated, safe in young children and pregnant women, and much cheaper than ACT. The efficacy of chloroquine to treat *P. vivax* malaria has not been evaluated in Mauritania. The present single-arm study was performed to evaluate the efficacy and tolerance of chloroquine in *P. vivax*-infected, symptomatic patients in the Saharan zone in Mauritania where *P. vivax* has emerged in recent years.

## Methods

### Study sites

**Figure 1:**
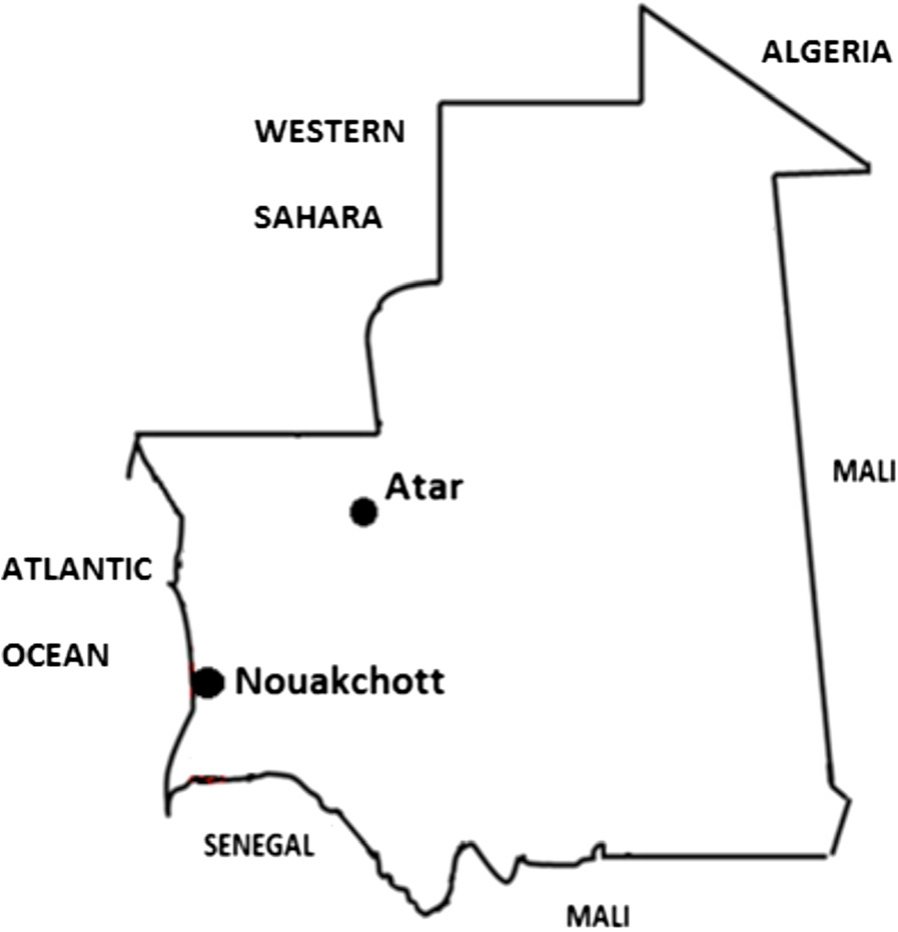
**Study sites in Mauritania.**

The clinical studies were conducted simultaneously in two study sites in central and northern Mauritania: Nouakchott (18°05′02″ north, 15°58′42″ west), the capital city of Mauritania, and Atar (20°31′06″ north, 13°03′13″ west, the regional capital of the Adrar) (Figure 1). In 2013, it was estimated that the population in Nouakchott was 899,887 inhabitants [Office National de la Statistique, Nouakchott, Mauritania]. Based on previous studies, that showed a high prevalence of laboratory-confirmed *P. vivax* malaria in the northern districts of Nouakchott [2,3], Teyarett health centre was selected for the present study. In 2012, the health centre recorded more than 1,000 *P. vivax* malaria, confirmed by rapid diagnostic test for malaria.

Atar lies within the Sahara desert and is situated 440 km to the northwest of Nouakchott. The estimated population of Atar in 2013 was 58,803 inhabitants [Office National de la Statistique, Nouakchott, Mauritania]. The study was conducted in the regional hospital with a functional basic laboratory. Although there are no previous malaria epidemiological data in Atar, a preliminary pilot study in 2012 showed laboratory-confirmed cases of *P. vivax* in this city [Ould Ahmedou Salem MS, unpublished data].

### Patients

Patients spontaneously consulting at the health centres in September–October 2013 were enrolled after informed written consent if the following criteria were met: age > 6 months old, *P. vivax* monoinfection with parasitaemia > 250 asexual parasites/μL, presence of fever (axillary temperature ≥ 37.5°C) or history of fever 48 h preceding medical consultation, and ability to swallow oral medication [12]. Patients with signs of severe and complicated *P. vivax* malaria (coma, impaired consciousness, respiratory distress, convulsions) [13,14], mixed *Plasmodium* species, severe malnutrition, fever due to concomitant diseases, and history of allergic reaction to chloroquine were excluded.

### Laboratory examinations, treatment, and follow-up

Fingerprick capillary blood was collected to prepare Giemsa-stained thin and thick smears to be examined under the microscope, according to standard World Health Organization (WHO) procedures [15]. Blood samples were also imbibed onto Whatman 3MM filter paper (GE Healthcare Life Sciences, Amersham Place, Little Chalfont Bucks, UK) and dried to store parasite DNA.

Chloroquine (batch no. CAMH0027 containing 240 mg chloroquine phosphate, an equivalent of 150 mg chloroquine base; Micro Labs Limited, Bangalore, India) was administered once daily on day 0 (10 mg base/kg body weight), day 1 (10 mg base/kg body weight), and day 2 (5 mg base/kg body weight) at the standard total dose of 25 mg base per kg body weight. Each dose administration was supervised by one of the team members, and patients were observed for at least 30 minutes after drug intake for possible vomiting or adverse effects. In case of vomiting during the observation period, the same dose was given, and the patient was observed for an additional 30 minutes. In case of repeated vomiting, the patient was withdrawn from the study protocol and rescue treatment was administered (parenteral quinine, 8 mg base/kg body weight, three times a day for seven days, or quinine followed by artesunate-amodiaquine or artemether-lumefantrine as soon as the patient tolerated oral medication). Paracetamol (10 mg/kg body weight) was administered to all patients three times a day for fever and headache.

Patients were followed on days 1, 2, 3, 7, 14, 21, and 28 [12]. Fingerprick capillary blood was collected for blood examination from day 2 to day 28 and imbibed onto Whatman 3MM filter paper from day 7 to 28, including any unscheduled visit due to an occurrence of fever, clinical aggravation, or treatment failure. Patients who failed to respond to chloroquine treatment were treated with artesunate-amodiaquine, as recommended by the national guideline for anti-malarial treatment.

### Study end points

The primary end point was determined on day 28 and classified into one of the following categories: early treatment failure (ETF), late clinical failure (LCF), late parasitological failure (LPF), or adequate clinical and parasitological response (ACPR), as defined by the WHO [12]. Treatment failure refers to one of the following outcomes: ETF, LCF, or LPF. ACPR is synonymous with treatment success.

### PCR and genotyping

Parasite DNA was extracted from filter papers using the Chelex method [16]. *Plasmodium* species identity was determined using 18S rRNA as the target molecule [17]. Genotypes of paired pre-treatment and recurrent parasites on or after day 7 were compared using microsatellite analysis [18]. At present, there is no standard protocol to distinguish among *P. vivax* recrudescence (i.e. reappearance of the same parasite population as that of pre-treatment infection), reinfection (i.e. appearance of different parasite populations), and relapse (reappearance of the same parasite populations or appearance of different parasite populations from reactivation of hypnozoites) with certainty.

### Statistical analysis

The sample size of 50 patients (plus 20% to anticipate losses due to exclusions, withdrawals, and lost-to-follow-up), i.e. a minimum of 60 patients, was calculated on the basis of an expected failure rate of 5%, 10% precision, and a confidence level of 95% [12].

As recommended in the WHO protocol [12], per protocol analysis of the percentage of ACPR and Kaplan-Meier survival analysis were performed to calculate the probability of the time to treatment failure during the 28-day follow-up period. Data were entered into the standard pre-programmed Excel worksheet provided by the Global Malaria Programme, WHO, for per protocol analysis. Survival curves were plotted and analyzed using Prism 4.0 (GraphPad Software, Inc., La Jolla, CA) software. Quantitative variables were compared between the patient population in Nouakchott and Atar using the unpaired *t*-test. The significance level was *P* < 0.05.

### Ethical approval

The study was reviewed and approved by an *ad hoc* Mauritanian national ethics committee and the ethics committee of the WHO (Geneva, Switzerland). The purpose of the study was explained to the patients (and their parents or legal guardians for children and adolescents) in local dialect. Informed written consent was obtained from adult patients or caretakers of paediatric patients aged less than 12 years old. For adolescents aged between 12 and 18 years old, an informed written consent was obtained from both the patients themselves and their parents or legal guardians.

## Results and discussion

A total of 128 patients were enrolled in the study: 67 in Nouakchott and 61 in Atar. Of 128 enrolled patients, 3 (2.3%, all in Nouakchott) were excluded after inclusion for protocol violation on day 1 (n = 1), withdrawal of consent on day 1 (n = 1), and development of urticaria on day 1 (n = 1) (Table 1). Additional six patients were lost-to-follow-up: two (both on day 28) in Nouakchott and four (on days 3, 7, 14, and 21) in Atar. Both patients who were lost to follow-up in Nouakchott (a 24-year-old male presenting with a body temperature of 41.2°C and 1,180 asexual parasites/μL on day 0, and a 35-year-old male with a body temperature of 37.8°C and 471 asexual parasites/μL on day 0) were afebrile from day 1 to day 21 and aparasitaemic from day 7. Among 4 patients lost to follow-up in Atar, one 40-year-old male presented with fever at the time of consultation (40.1°C with 1,600 asexual parasites/μL), and 3 (5, 24, and 28-year old patients) had a history of fever less than 48 h preceding medical consultation and parasitaemia ranging from 600 to 3,300 asexual parasites/μL. All of these four patients were afebrile from day 1 and aparasitaemic from day 2 until the last day when they were seen. The per protocol population consisted of 119 patients followed until day 28, 62 in Nouakchott and 57 patients in Atar. PCR showed that all included patients were infected by *P. vivax*.

**Table 1:** **Characteristics of** ***P. vivax*** **-infected patients on inclusion**

**Characteristics**	**Nouakchott**	**Atar**
Number of enrolled patients	67	61
Mean age (±SD, range), yr	23.9 ± 16.5 (3–62)	30.9 ± 17.9 (2–80)
Age groups		
Under 5 years old, n (%)	5 (7.5)	2 (3.3)
5 – 18 years old, n (%)	25 (37.3)	17 (27.9)
>18 years old, n (%)	37 (55.2)	42 (68.9)
Sex ratio (M/F)	0.91 (32/35)	0.85 (28/33)
Mean weight (±SD, range), kg	49.2 ± 24.4 (9–98)	54.4 ± 21.1 (10–102)
Mean body temperature (±SD, range), °C	38.8 ± 1.2 (36.0–41.2)	39.0 ± 1.5 (36.0–41.6)
Proportion of patients with fever (≥37.5°C) (%)	92.5	70.5
Geometric mean parasitaemia (95% confidence interval; range), asexual parasites/μL	988 (834–1 170)	2 280 (1 850–2 810)
	290–14 300	460–30 100

The mean age and parasitaemia were significantly higher (*P* < 0.05) in Atar than in Nouakchott. There was no significant difference (*P* > 0.05) in the mean weight and body temperature of the two patient populations in Nouakchott and Atar.

In both Nouakchott and Atar, per protocol analysis showed 100% chloroquine efficacy (Table 2). Due to the absence of treatment failure, PCR correction using microsatellites and survival curve analysis were not performed.

**Table 2:** **Per-protocol analysis of chloroquine efficacy to treat** ***P. vivax*** **malaria in the Saharan zone in Mauritania**

**Outcome**	**Nouakchott**	**Atar**
Number of enrolled patients	67	61
Exclusion after enrollment, n (%)	3 (4.5)	0
Lost-to-follow-up, n (%)	2 (3.0)	4 (6.6)
Per protocol population, n	62	57
PCR-uncorrected outcome*		
Early treatment failure, n (%)	0	0
Late clinical failure (LCF), n (%)	0	0
Late parasitological failure, n (%)	0	0
Adequate clinical and parasitological response, n (%)	62 (100)	57 (100)

*PCR correction was not performed due to 100% efficacy (per protocol population) on day 28.

This is the first evaluation of chloroquine efficacy against *P. vivax* in West Africa. The results indicate that chloroquine is highly efficacious and well-tolerated in the predominantly Moor population residing in Nouakchott and Atar. Fever and parasitaemia were rapidly cleared. Generalized urticaria developed 2–3 hours after the first dose of chloroquine in a 7-year old boy. The patient was immediately treated with injectable dexamethasone and oral antihistaminics, and the patient was excluded from the protocol by precaution and treated with artemether-lumefantrine.

Although chloroquine was found to be highly effective in the present study, chloroquine-resistant *P. vivax* has been reported from some foci in Asia, Oceania, South America, and more recently, from Madagascar and Ethiopia in eastern Africa [11,19-23]. In the most recent study conducted in Ethiopia, recurrent parasitaemia was observed in 10 (9.3%) of 108 enrolled patients (per protocol population) on day 28 [23]. Three of 10 cases of recurrent parasitaemia had identical genotype as the day-0 parasites. Furthermore, in that study recurrence was observed in 32% of patients treated with chloroquine by day 42. However, despite reappearance of parasite populations with identical genotype as the pre-treatment parasites, the origin of recurrent *P. vivax* parasitaemia after treatment cannot be attributed at present to recrudescence, reinfection, or relapse with certitude due to technical limitations. A co-administration of chloroquine and primaquine may reduce the recurrence rate due to relapse.

A nationwide survey to establish an up-to-date epidemiology of glucose-6-phosphate dehydrogenase deficiency, which presents a potential risk of primaquine-induced haemolysis, and clinical studies on chloroquine co-administered with primaquine are warranted to further assess and determine the role of chloroquine for the treatment of *P. vivax* malaria in Mauritania.

## Conclusions

More precise, up-to-date epidemiological data on the distribution of *P. vivax* and glucose-6-phosphate dehydrogenase deficiency in Mauritania are required before implementing concrete actions to control and eliminate malaria from the country. Based on the results of the present clinical data, the current national treatment guideline that recommends the systematic use of artesunate-amodiaquine for the first-line treatment of acute uncomplicated *P. falciparum* and *P. vivax* malaria in Mauritania may need to be revised if further studies confirm chloroquine efficacy in other sites in the country.

## Abbreviations

ACPR: Adequate clinical and parasitological response
ACT: Artemisinin-based combination therapy
ASAQ: Artesunate-amodiaquine
ETF: Early treatment failure
LCF: Late clinical failure
LPF: Late parasitological failure
PCR: Polymerase chain reaction
